# Stroke From Paradoxical Embolism in a Young Patient With Sickle‐Cell Disease and High Fetal Hemoglobin: A Diagnostic Challenge

**DOI:** 10.1002/ccr3.72618

**Published:** 2026-04-24

**Authors:** Yi Hui Luo, Navid Zuberi, Angelo Rizzolo, Margaret Warner, Stephane Isnard, Jean‐Pierre Routy

**Affiliations:** ^1^ Department of Internal Medicine McGill University Montreal Quebec Canada; ^2^ Division of Hematology McGill University Health Centre Montreal Quebec Canada; ^3^ Research Institute of the McGill University Health Centre Montreal Quebec Canada

**Keywords:** fetal hemoglobin, hemoglobin S, sickle cell disease, stroke

## Abstract

Sickle‐cell disease (SCD) is characterized by abnormal hemoglobin (Hb) polymerization, leading to erythrocyte sickling and microvascular obstruction. Elevated fetal hemoglobin (HbF) levels are often associated with reduced—but still possible—vaso‐occlusive complications. We report the case of a 22‐year‐old South Asian male with SCD and persistently elevated HbF (~23%) who presented with transient right‐sided numbness and visual disturbances. Brain MRI confirmed acute ischemic infarcts, prompting urgent red blood cell exchange transfusion. Comprehensive investigations for causes of stroke revealed a patent foramen ovale on echocardiogram, suggesting paradoxical embolism as the etiology. This case highlights diagnostic challenges in SCD patients with high HbF presenting with stroke. While elevated HbF levels are considered protective against vaso‐occlusive events, this patient's presentation challenged this assumption. The identification of a cardiac shunt prompted reconsideration of the stroke mechanism. In conclusion, clinicians must maintain a broad differential diagnosis and pursue comprehensive evaluations in SCD patients with neurological symptoms.

## Key Clinical Message

High fetal hemoglobin (HbF) levels are considered a protective factor against vaso‐occlusion during sickle cell disease (SCD). However, this case illustrates the diagnostic challenge of a stroke in a young patient with high HbF, leading to structural heart defect identification, warranting complete cardiovascular evaluation in all SCD patients.

## Graphical abstract

Acute infarcts and microvascular ischemic changes in the brain of a young patient with Sickle‐Cell Disease despite High Fetal Hemoglobin.

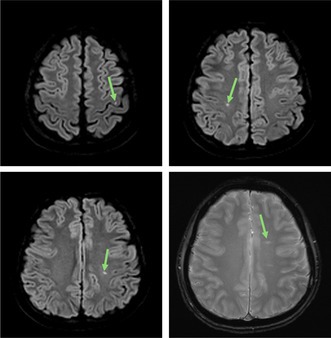

## 1 Introduction

Sickle‐cell disease (SCD) is the commonest hereditary hemoglobinopathy. It results from abnormal hemoglobin S (HbS) polymerizing under hypoxemic states, causing red blood cells (RBCs) to adopt a sickle shape. Its pathophysiology encompasses chronic hemolysis and microvascular occlusions. Conditions causing high levels of fetal hemoglobin (HbF), such as hereditary persistence of fetal hemoglobin (HPFH) or certain beta‐globin gene haplotypes in SCD are protective against vaso‐occlusive complications, although severe complications are still possible [1].

Risk of stroke is elevated in SCD patients and requires urgent treatment with RBC‐exchange transfusion to rapidly decrease the fraction of HbS [2]. Furthermore, stroke occurring in a young patient must prompt a comprehensive search for causes including arrhythmia, cardiovascular disease, structural heart defects, and procoagulant states.

## 2 Case History/Examination

A 22‐year‐old male of South Asian descent presented to the emergency department with a first‐episode 1‐day history of intermittent right hemibody heaviness and numbness, and an expanding dark area in his visual field. Episodes lasted approximately 20 min each and spontaneously resolved without sequelae. He was asymptomatic at the time of presentation.

The patient is a student who does not smoke nor drink alcohol, with a family medical history notable for a cousin with SCD. At age 20, he presented with hematuria. Abdominal magnetic resonance imaging (MRI) showed unremarkable kidneys, but the presence of splenic infarcts raised suspicion of SCD. Peripheral blood smear confirmed the presence of sickle cells and Hb electrophoresis showed HbS at 70.7%, baseline elevated HbF at 27.6%, and HbA2 at 1.7%. He was considered to have either HPFH or an HbS haplotype with baseline elevated HbF. He had never received hydroxyurea. He had very rare vaso‐occlusive crises (VOC) and had never presented with acute chest syndrome, stroke, or hepatic or infectious complications.

## 3 Differential Diagnosis, Investigations and Treatment

**FIGURE 1:**
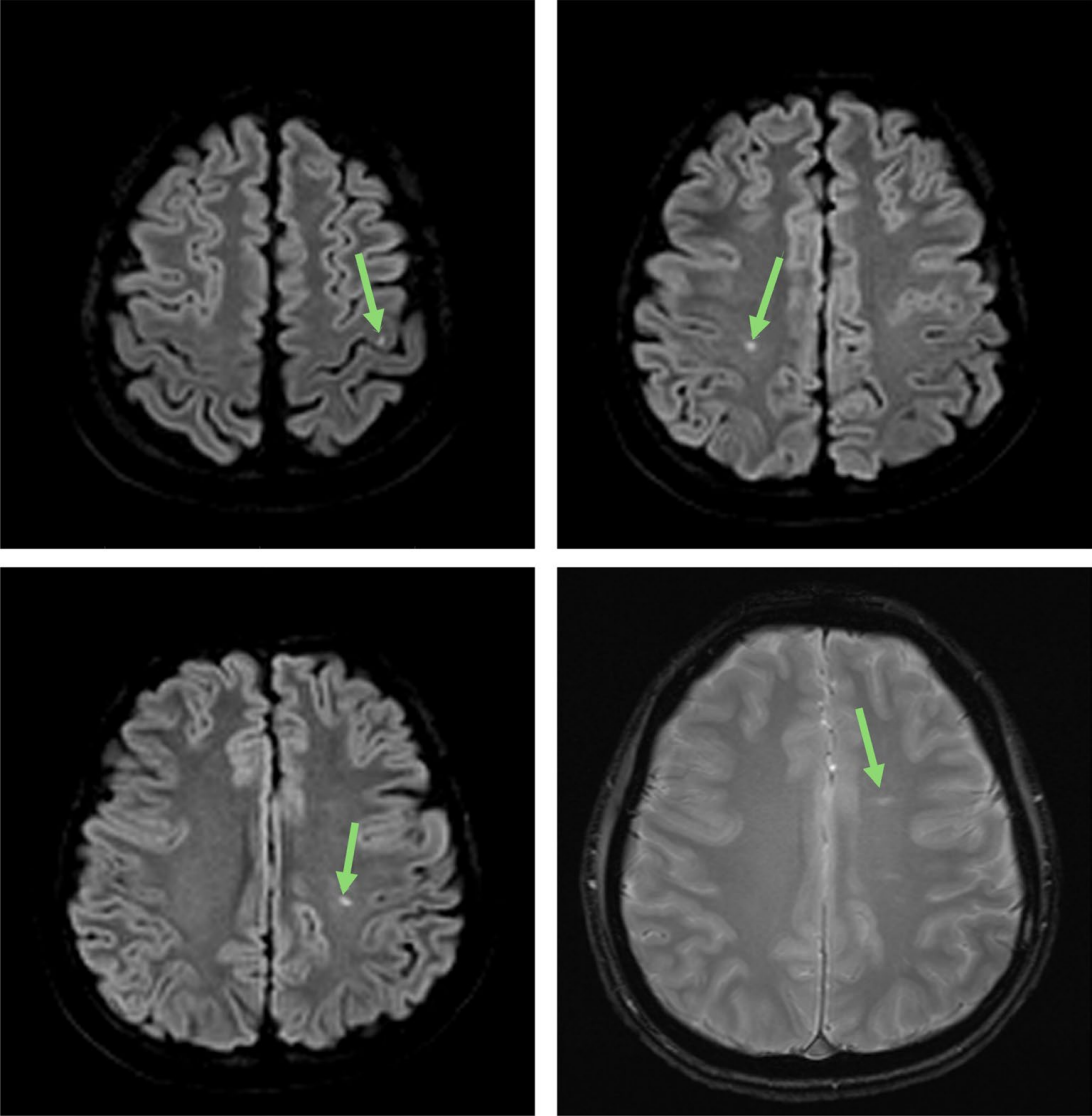
Brain MRI showing three foci of acute infarcts (top two images and bottom left, DWI sequence) and signs of early chronic microvascular ischemic changes (bottom right, T2/FLAIR sequence).

Review of systems was negative for infection, headache, syncope, chest pain, dyspnea, digestive and urinary symptoms, musculoskeletal pain, and lower extremity edema. He had no focal neurological deficit. Pertinent laboratory investigations are listed in Table 1. Brain MRI showed three foci of acute infarcts and signs of early chronic microvascular ischemic changes in the left frontal centrum semiovale (Figure 1).

**TABLE 1:** Relevant laboratory investigations during initial workup.

Parameters	Value	Laboratory reference
Hemoglobin (g/L)	122	135–175
Mean cell volume (fL)	93.6	80–100
Absolute reticulocytes (10^9^/L)	267.6	20–120
C‐reactive protein (mg/L)	1.8	0–5
International normalized ratio	1.07	0.88–1.12
Creatinine (μmol/L)	54	55–110
LDL cholesterol (mmol/L)	0.84	
Apolipoprotein B (g/L)	0.41	0–0.8
Hemoglobin A1c	5.9%	4.3–6.0
Ferritin (μg/L)	62.3	23.9–366
Beta‐2‐glycoprotein (units/mL)	< 1.4	
Anticardiolipin IgM and IgG	Negative	
Lupus anticoagulant	Negative	
Protein C (units/mL)	0.95	0.72–1.56
Protein S free (units/mL)	0.49	0.55–1.25
Factor V assay (units/mL)	1.01	0.50–1.50
Antithrombin III assay (units/mL)	0.91	0.81–1.19
APC resistance (s)	192.8	120.3–222.2
High performance liquid chromatography		
Hemoglobin A2	2.7%	1–3.5
Hemoglobin F	21.2%	≤ 2
Hemoglobin A	1.5%	
Hemoglobin S	75.4%	

The patient underwent urgent RBC‐exchange transfusion to treat suspected stroke related to SCD. The parameters were the following: 105% fluid balance, 3375 mL given in packed RBC units, yielding a target hematocrit of 31% and a fraction of cells remaining at 26%.

**FIGURE 2:**
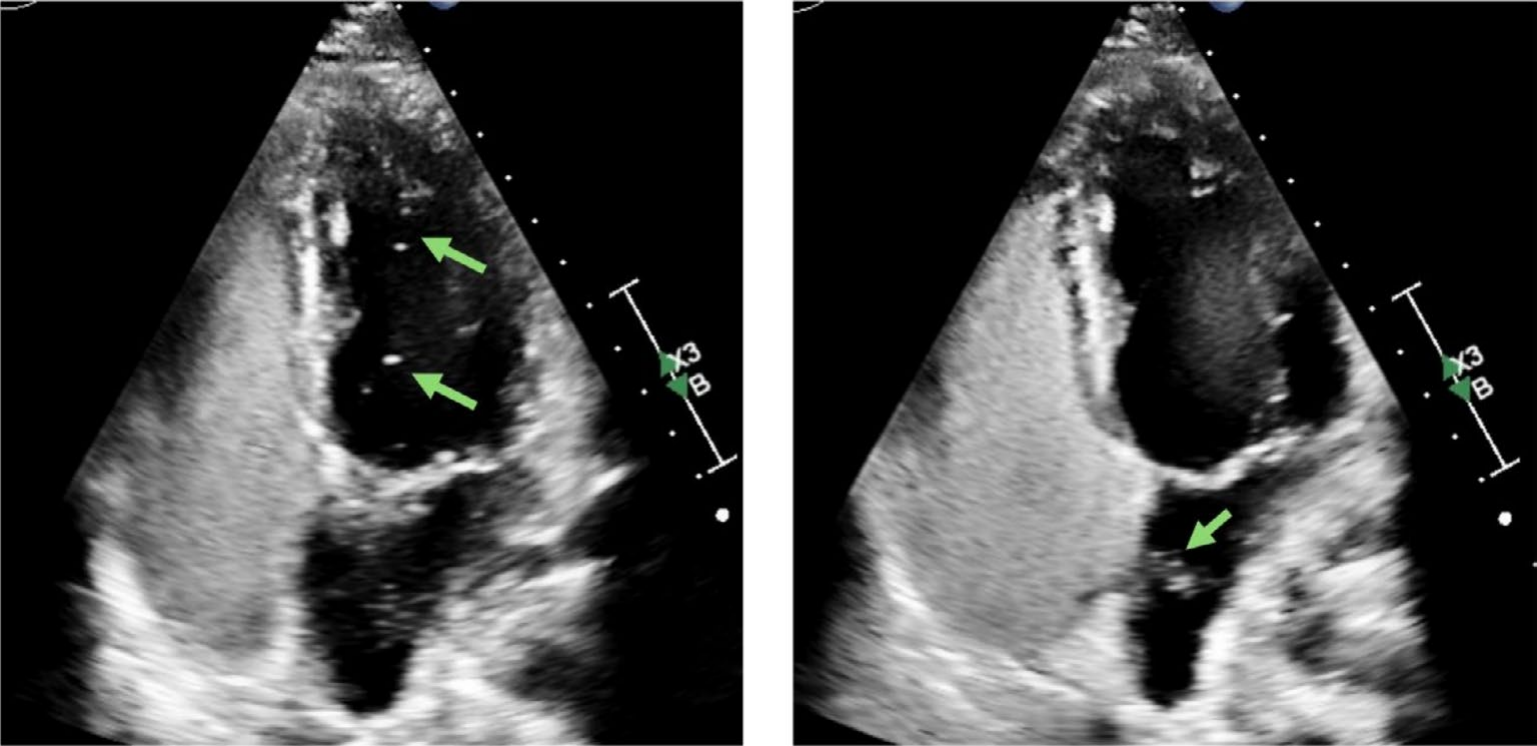
Bubble study demonstrating passage of bubbles into the left heart (left) via right‐to‐left shunting through the PFO (right) on transthoracic echocardiogram.

**FIGURE 3:**
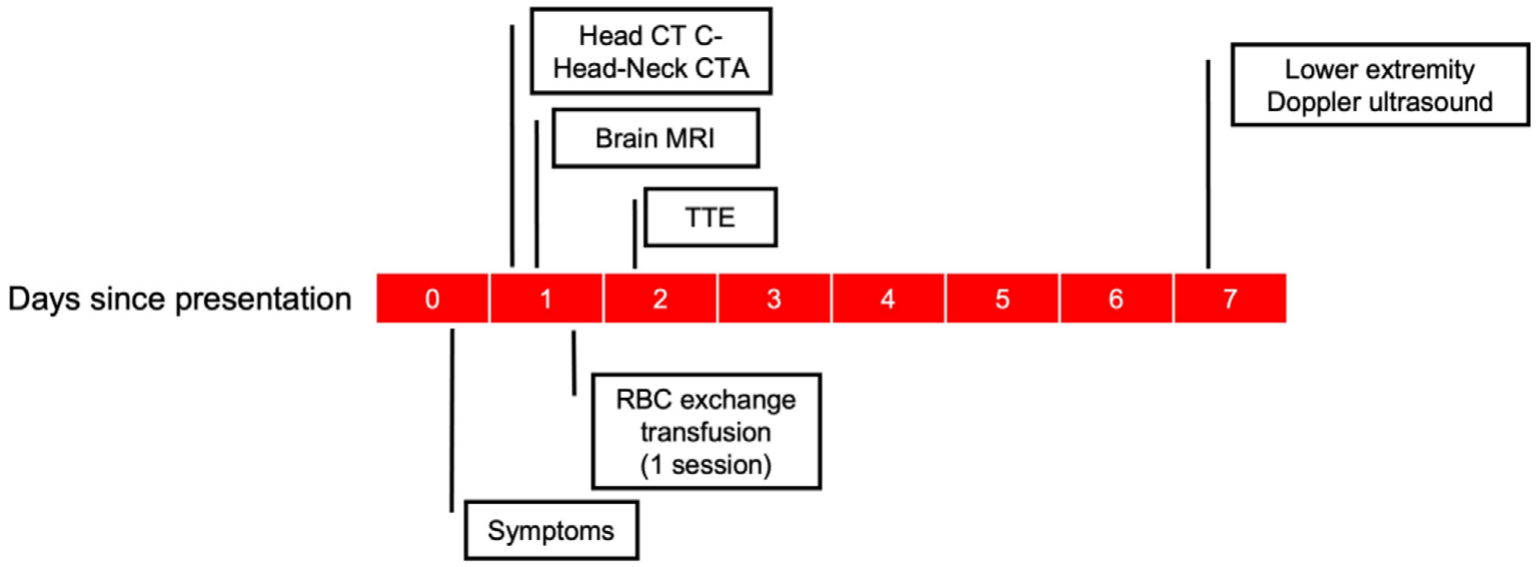
Timeline of investigations and exchange transfusion during initial admission. Day 0 was the only day of symptoms. CTA, CT angiography.

Given this unexpected neurological presentation in a young patient with presumed protection from high HbF, causes of stroke unrelated to SCD were investigated. He had no metabolic comorbidities. Thrombophilia workup was negative (Table 1). CT angiography showed patent cervical and intracranial arteries. Transthoracic echocardiogram with bubble study revealed a patent foramen ovale (PFO) with right‐to‐left shunting (Figure 2). Admission ECGs and outpatient Holter monitoring were negative for atrial fibrillation. While lower extremity Doppler ultrasound showed no deep venous thrombosis (DVT), this did not exclude prior silent DVT (Figure 3).

## 4 Conclusion and Results

Clinical scores suggested a possible causal relationship between the PFO and stroke in our patient (RoPE score was 88% and PASCAL classification suggested possible causality). The patient did not present with hypertension, obesity, dyslipidemia, or smoking. Echography of the heart excluded endocarditis, including Libman‐Sacks or cardiac myxomas. The protective effect of high HbF in this patient, the finding of a PFO in absence of alternate causes, and suspicion of PFO‐associated stroke suggested paradoxical embolic stroke as the cause of his symptoms.

Per consulting neurologist recommendation, he started daily aspirin 80 mg orally for secondary prevention and there was no indication for therapeutic anticoagulation. He subsequently underwent atrial septal defect patching to prevent future paradoxical embolism. At 9‐month follow‐up, repeat brain MRI showed no new lesions and the patient was well with no recurrence of vaso‐occlusive crises nor stroke.

## 5 Discussion

Heterozygous HbS carriers typically have no symptoms of vaso‐occlusive pain except under extreme circumstances. Patients with homozygous beta‐globin mutations for HbS (HbSS), those with HbSC, HbS‐beta thalassemia, and other combinations with hemoglobin variants have more severe phenotypes (SCD). High levels of HbF decrease the concentration of HbS in blood, minimizing HbS‐induced sickling and VOC [1]. Increasing HbF levels is one of the mechanisms of action of hydroxyurea in patients with SCD.

Adults with HbS continue producing HbF at elevated levels depending on several factors, including their ethnic origin. Individuals with the Arab‐Indian β‐globin gene typically have higher HbF levels, which are sometimes associated with less severe SCD course, as compared to people with the African haplotype [1, 3]. Studies from India have shown mean HbF levels of 7.9%–23.7% in patients with SCD and 1%–1.4% in patients with sickle trait [4, 5]. As our patient originates from India, we suspect that he carries the Arab‐Indian haplotype.

HPFH is a rare and heterogeneous group of conditions in which genetic mutations lead to increased production of HbF well past the first year of life. Mechanistically, HbS‐HPFH causes uniformly high, pancellular HbF expression that prevents sickling, whereas high‐HbF haplotypes show uneven HbF distribution, leaving some RBCs vulnerable. Thus, patients with high‐HbF haplotypes have more frequent sickle cell‐related complications than those with HPFH mutation [6, 7]. Individuals with HbS and concomitant inheritance of HPFH (HbS‐HPFH) may have unusually high HbF levels [6]. While genetic testing may help predict disease severity or inform genetic counseling, whether it influences management beyond conventional testing is individually determined.

HbS‐HPFH patients typically have fewer vaso‐occlusive complications [6, 8], though few genotype‐confirmed studies exist. Still, case reports describe severe SCD complications in some HPFH patients [9]. Painful bone infarcts, pulmonary disease, severe acute splenic sequestration, and massive splenic infarction have also been described. While the exact incidence of complications is unclear, these cases demonstrate that patients with HbS‐HPFH are not immune to acute life‐threatening complications, for which maintaining a high degree of suspicion is warranted.

This case illustrates the diagnostic challenge of stroke in a patient with SCD and high HbF, typically linked to low vaso‐occlusive risk. It emphasizes the need for a thorough differential diagnosis and comprehensive evaluation, which in this instance revealed a PFO as the likely cause of stroke and significantly influenced management as our patient underwent PFO closure [10]. Improved risk stratification models would allow for better prevention of cerebrovascular complications in SCD patients.

## Author Contributions

**Yi Hui Luo:** conceptualization, data curation, formal analysis, investigation, methodology, visualization, writing – original draft, writing – review and editing. **Navid Zuberi:** data curation, formal analysis, investigation, writing – review and editing. **Angelo Rizzolo:** data curation, formal analysis, investigation, writing – review and editing. **Margaret Warner:** conceptualization, investigation, supervision, writing – review and editing. **Stephane Isnard:** writing – review and editing. **Jean‐Pierre Routy:** conceptualization, investigation, supervision, writing – review and editing.

## Funding

The authors have nothing to report.

## Ethics Statement

The authors have nothing to report.

## Consent

The patient provided written informed consent for the preparation of this case report and publication of data and pathological image in an open access journal.

## Conflicts of Interest

J.‐P.R. is an invited speaker for Recordati rare diseases. The other authors declare no conflicts of interest.

## Data Availability

The data that support the findings of this study are available from the corresponding author upon reasonable request.
